# Modeling the Complex Impacts of Timber Harvests to Find Optimal Management Regimes for Amazon Tidal Floodplain Forests

**DOI:** 10.1371/journal.pone.0136740

**Published:** 2015-08-31

**Authors:** Lucas B. Fortini, Wendell P. Cropper, Daniel J. Zarin

**Affiliations:** 1 U.S. Geological Survey Pacific Island Ecosystems Research Center, Honolulu, Hawaii, United States of America; 2 School of Forest Resources and Conservation, University of Florida, Gainesville, Florida, United States of America; 3 Climate and Land Use Alliance, San Francisco, California, United States of America; Chinese Academy of Forestry, CHINA

## Abstract

At the Amazon estuary, the oldest logging frontier in the Amazon, no studies have comprehensively explored the potential long-term population and yield consequences of multiple timber harvests over time. Matrix population modeling is one way to simulate long-term impacts of tree harvests, but this approach has often ignored common impacts of tree harvests including incidental damage, changes in post-harvest demography, shifts in the distribution of merchantable trees, and shifts in stand composition. We designed a matrix-based forest management model that incorporates these harvest-related impacts so resulting simulations reflect forest stand dynamics under repeated timber harvests as well as the realities of local smallholder timber management systems. Using a wide range of values for management criteria (e.g., length of cutting cycle, minimum cut diameter), we projected the long-term population dynamics and yields of hundreds of timber management regimes in the Amazon estuary, where small-scale, unmechanized logging is an important economic activity. These results were then compared to find optimal stand-level and species-specific sustainable timber management (STM) regimes using a set of timber yield and population growth indicators. Prospects for STM in Amazonian tidal floodplain forests are better than for many other tropical forests. However, generally high stock recovery rates between harvests are due to the comparatively high projected mean annualized yields from fast-growing species that effectively counterbalance the projected yield declines from other species. For Amazonian tidal floodplain forests, national management guidelines provide neither the highest yields nor the highest sustained population growth for species under management. Our research shows that management guidelines specific to a region’s ecological settings can be further refined to consider differences in species demographic responses to repeated harvests. In principle, such fine-tuned management guidelines could make management more attractive, thus bridging the currently prevalent gap between tropical timber management practice and regulation.

## Introduction

One underlying tenet of sustainable forest management (SFM) is that forests should provide for multiple needs continuously into the future [1]. For remote tropical areas where timber is a main economic resource, sustainable timber management (STM; which aims to ensure continued timber yields) should be an important component of SFM ([2,3] but see [4]). In the Amazon, the results of most research on STM has been discouraging [5–7]. In the Amazon estuary, however, the persistence of forests after centuries of harvests suggests STM potential [8,9].

At present, hundreds of small-scale, family-run timber operations in the Amazon estuary are supplied by many more thousands of smallholders that rely on timber as a source of income [10,11]. These production systems have been characterized as both sustainable [12,13] or unsustainable [14]. Recent research has explored the links between forest composition, tree population ecology and the long history of selective logging in the region [15,16]. However, the extent to which tidal floodplain tree demography affects post-harvest recovery and, consequently, the prospects for STM still remains unclear.

Most research on the impacts of timber harvest/management on tropical forests have focused on either harvest damage, post-harvest effects on growth, recruitment and mortality independently [17–19]. Hence, the integrated effects of timber harvest and management on population demographic responses are poorly known for most tropical forests. While some models have been developed to address the long-term ecological impacts of timber harvest and management [6,20–22], these have largely focused on evaluating current practices rather than identifying or assessing sustainable management alternatives.

We used a matrix-based management simulation model to evaluate the prospects for sustained timber production in the tidal floodplain forests of the Amazon estuary. Matrix population models are often used to simulate long-term impacts of forest harvest and management [23]. However, matrix-based management models often simulate harvests by simply ‘vanishing’ harvestable trees from the projected population distributions [24]. This approach ignores the common impacts of tree harvests including incidental damage, changes in post-harvest demography, shifts in the distribution of merchantable trees, and shifts in stand composition. We used field data from harvest monitoring and harvested inventory plots to create a matrix-based management simulation model that integrates the simulation of all of these aspects of timber harvest into models that explore the long-term outcomes of hundreds of possible management regimes for forests of the Amazon Estuary. Based on these results, we find optimal stand-level (all modeled species combined) and species-specific sustainable timber management regimes relevant to forests in the Amazon Estuary.

## Methods

### Study region and species

We conducted our research in the 160 km^2^ Mazagão watershed at the Western side of the Amazon estuary (-0.26°N, -51.35°E) characterized by freshwater tidal fluctuations of 2–3 m. This area has a long history of small-scale timber use [12]. We chose 8 timber species that account for the majority of merchantable volume extracted in the region over the past century as well as species that are likely to have timber value in the future. *Platymiscium filipes*, *Carapa guianensis*, and *Virola surinamensis* were extracted during the logging boom from the 1950s until the 1990s [12]. Since then, *Callycophyllum spruceanum*, *C*. *guianensis*, *V*. *surinamensis*, *and Licaria mahuba* have been the primary species utilized in the region [16]. *Mora paraensis*, a species with high density wood, has only been harvested at commercial levels by smallholders over the last decade because its weight and low buoyancy make ground and water transport difficult. *Licania heteromorpha* and *Pouteria sagotiana* are only occasionally harvested but were nonetheless included in the projections. All research was conducted within private properties with all owners’ consent. As no human subjects were part of the research, the University of Florida IRB office deemed no IRB approval was necessary.

### Permanent inventory plots

Species demography from recently undisturbed forests (stands with no sign of recent timber harvests) was estimated from three 360 x 360 m plots (13 ha per plot, 39 ha total; unharvested plots *hereafter*) established and monitored yearly from 2005 to 2008 and five 1 ha plots first measured in 1997 and then yearly from 2004 to 2007. We established 14 small permanent inventory plots (totaling 6.2 ha) in areas logged 1–6 years before plot establishment (harvested plots *hereafter*). Due to the small scale of logging operations [25], harvested plots were either 60 x 60 m or 80 x 80 m to avoid surrounding unharvested areas. Using the same methodology for unharvested plots [16], we measured diameter, growing condition and stem form of all trees > = 5 cm DBH within all harvested plot trees in 2007 and 2008. A total of 5800 trees were monitored for this study.

To avoid the unrealistic yield-inflating assumption that all harvest-sized trees are merchantable [26], all inventory trees were classified into merchantable/ unmerchantable grade by a crew with expertise in local logging practices. We defined unmerchantable trees as those with defects severe enough that they would not be felled. Lastly, commercial height (i.e., height of crown base) was estimated using vertical hypsometers.

### Harvesting damage assessment

In July–August 2008 we monitored the harvesting activities of 3 local logging crews to evaluate residual stand damage and related logging practices. A total of 413 man-hours were monitored in which the extraction of 40 standing trees was followed from forest to sawmill. We identified the species and measured the DBH of all trees harvested, damaged or killed during logging. Because damaged trees may not die immediately following harvests, trees with extreme harvest damage were assumed dead [27].

### Management simulation model

To simulate the impact of continued timber harvests on tree populations, we expanded matrix models developed in Fortini and Zarin [16] that projected population dynamics of all study species in recently undisturbed forest settings. The updated model uses data from the harvested plots, harvest damage assessments, and merchantable tree surveys to evaluate a suite of long-term management regimes defined by varying harvest rotation length, species harvested, minimum cut diameter (MCD), harvest intensity (proportion of trees with DBH ≥ MCD harvested, with the remaining left as seed trees), minimum density (MD) and limits of harvest volume per ha. Model mechanics are described in detail below.

#### Modeling unmerchantable stems

Merchantable and unmerchantable classes of each species were modeled as distinct but interacting populations [28] to reflect size-dependent shifts in merchantable/ unmerchantable population ratios (Table 1). We used only data from seldom-harvested *M*. *paraensis* to estimate the projected transition rate of individuals from merchantable to unmerchantable status with increasing size. Despite potential differences among species in this merchantable/ unmerchantable ratio, the use of *M*. *paraensis* alone was preferred to reduce biases resulting from past undocumented harvests in the region. While this approach may lead to optimistic first harvests for species more heavily used in the past, it yields a clearer pattern of increased incidence of defects with size which is more important when projecting long-term population dynamics beyond a first harvest.

**Table 1:** Transition matrix model used for management simulations. *x-* Probability that merchantable (M) trees growing to next size class will become unmerchantable (UM) because of defects. S = survival probability, G = size class upgrowth probability, F = Fertility rate per capita; Number of DBH size classes (denoted by subscripts) are reduced for better visualization as size classes are 2.5 cm wide, except for first size class (5–9.9 cm DBH).

		T									
		M_1_	M_2_	M_3_	M_4_	M_5_	UM_1_	UM_2_	UM_3_	UM_4_	UM_5_
**T+1**	**M**_**1**_	S_1_		F_3_	F_4_	F_5_			F_3_	F_4_	F_5_
	**M**_**2**_	G_1_	S_2_								
	**M**_**3**_		G_2_*(1-x_2_)	S_3_							
	**M**_**4**_			G_3_*(1-x_3_)	S_4_						
	**M**_**5**_				G_4_*(1-x_4_)	S_5_					
	**UM**_**1**_						S_1_				
	**UM**_**2**_						G_1_	S_2_			
	**UM**_**3**_		G_2_*(x_2_)					G_2_	S_3_		
	**UM**_**4**_			G_3_*(x_3_)					G_3_	S_4_	
	**UM**_**5**_				G_4_*(x_4_)					G_4_	S_5_

Our model simulates the irreversible transition of merchantable individuals into unmerchantable status due to the development of stem defects. Simulated harvests include only merchantable trees and thus result in a shift in the merchantable/ unmerchantable population ratios. Unmerchantable trees are still projected each year, contribute to yearly regeneration and density dependent population regulation, and experience residual stand damage at each harvest (*see below*). Lastly, as a management criteria, seed-tree selection can include the preferential selection of unmerchantable trees (possibly leading to low seed-tree quality, assuming short-term profit maximization behavior by harvester) or may include only the merchantable proportion of the population (high seed-tree quality).

#### Modeling density dependence

We included density dependent recruitment regulation [29] using a complemented Weibull function [30] to avoid unrealistic model outcomes due to the intrinsic exponential nature of matrix projections [31].

$${Recruitment}_{Na}=a\times exp{\left(\frac{-Na}{b}\right)}^{6}$$

Where *a* and b are parameters related to density at carrying capacity (K) and Na is current stand density per ha. The complemented Weibull function was chosen because it responds slowly to increased density at populations far from K, but regulates recruitment rates at an increasing rate as densities approach K, as opposed to other commonly used models [32].

K was calculated as the maximum subplot population density observed in all data from our unharvested plot data. This calculation was done through a 100 x 100 m moving window algorithm that avoided effects from small-scale spatial variability in tree distribution. We used species-specific Ks that were scaled proportionally to the maximum density observed for all species combined since it is highly unlikely that the observed highest densities for all species would be observed in a same subplot due to limits on overall stand density. We also experimentally configured our density dependent model to consider stand level density across all modeled species as a way to incorporate species composition shifts due to continued harvests (S1 File).

#### Harvest damage modeling

Based on field data, we calculated a residual stand mortality ratio [trees incidentally killed:trees harvested]. After each simulated harvest, we applied the ratio to all populations based on the number of trees harvested. Because we found no clear size-related patterns in residual stand mortality, the residual stand mortality ratio used in the model is indiscriminate of merchantable and unmerchantable trees and size class.

#### Post-harvest demographic effects assessment and modeling

All trees surviving a harvest are modeled using matrices that include post-harvest demographic effects. First, we attempted to define the length of post-harvest effects on growth using standard least square regression of post-harvest diameter increments against years since logging. This analysis revealed no clear impact of time since harvest on growth across harvested plots, suggesting post-harvest effects last longer than the amount of time between harvest and monitoring in our harvested plots. Based on limited literature indicating that post-harvest effects on growth generally do not last longer than 10 years [33,34], we chose to simulate a post-harvest effect duration of 10 years in our models. Because we detected no effect of time since last harvest in the harvested plot demography data, we grouped all harvested plots together for subsequent analyses. With these pooled data, we determined post-harvest demographic effects as the proportional change in diameter growth, recruitment, and mortality rates between unharvested and harvested plots for the 2007–2008 measurement interval. We then created post-harvest projection matrices for each species by altering the matrices created from unharvested plot data [16] using the observed post-harvest demographic effects.

Besides using harvested plot data to modify unharvested plot matrices to project population dynamics post-harvest, we computed treatment differences in individual/ environmental factors that could influence demography including flooding and light regimes [16] to explore the causes of post-harvest effects on growth and a life table response experiment (LTRE) analysis to determine which vital rates contributed most to differences in population growth between harvested and unharvested plots [28,35,36].

#### Management simulation model outputs

Every management simulation included a projection period of 120 years. Population annual growth rates (λ) under simulated harvest regimes were calculated empirically from model projections.

$$\lambda_{H}={\left(\frac{N_{yr120}}{N_{yr0}}\right)}^{\frac{1}{120}}$$

The model also calculated the necessary compensatory recruitment needed to be planted yearly to stabilize population size at pre-harvest levels.

#### Simulation of multiple management regimes

Each of the 1440 management regimes we simulated represents combinations of possible management criteria: cutting cycles of 10–40 years; MCDs of 30–70 cm DBH; harvest intensity of 0.5–0.9 of stand volume; minimum species density (MD) of 0.03 and 1 trees/ha per harvest species; high and low seed-tree quality (i.e., exclusion or inclusion of unmerchantable trees in the calculation of harvest intensity); and simulations with or without a harvest volume limit of a 1 m^3^ ha^-1^ yr^-1^ (Table 2). The range of management regimes simulated also included the Brazilian legal management regimes (i.e., 10 or 30 yr cutting cycle, maximum 90% harvest intensity, 50 cm MCD, MD of 0.03 trees/ha and a 1 m^3^ ha^-1^ yr^-1^ harvest volume limit; Table 2; http://ibama2.ibama.gov.br). Each of these management regimes was applied for the duration of the 120 yr projection period to a hypothetical 1ha forest stand that represents the average size distribution for each species across all surveyed plots.

**Table 2:** Values for management criteria used in management simulations.

Cutting cycle	Harvest intensity	MDC	Min. com. density	Seed tree quality	Max volume per harvest (m^3^ yr^-1^ ha^-1^)
10*	0.5	30	0	High	-
20	0.6	40	0.03*	Low*	1*
30*	0.7	50*	1		
40	0.8	60			
	0.9*	70			

* denote criteria specified by Brazilian law

To evaluate which management regimes produced the best long-term yield and least long-term population impacts, all management regime simulation outcomes were rated according to 4 different STM indicators: annualized yield of fifth harvest (H_5_ AY), annualized yield of third harvest (H_3_ AY), mean annualized yield (mean AY) during the 120 years excluding the first harvest, and the summation of the ranked mean annualized yield and the ranked λ_H_ (ranked AY and λ_H_). First harvests were excluded from mean AY because annualized yields are not computable.

To evaluate inter-specific differences in management regimes outcomes, all optimal species-specific management regimes were computed. This allowed for a comparison to determine whether differences in species-specific optimum management regimes led to suboptimal management at the stand level.

## Results

### Post-harvest demographic effects

Nearly all species showed a positive diameter growth response after logging. Surprisingly, *C*. *spruceanum*, the most shade-intolerant species in the study, was the only one that showed a small diameter growth decrease in response to logging. Most species showed a proportional increase in growth rates of trees in the juvenile size class (5–20 DBH) and no consistent treatment response for the adult size classes (>20 DBH). Hence, we classified species post-harvest growth effects into juvenile and adult values. Analysis of individual tree / environmental factors that could influence demography revealed that a larger proportion of 5–20 cm DBH trees were observed under high light conditions in harvested plots compared to unharvested plots (25% vs 7%, respectively). Only *Mora paraensis* showed a recruitment boost into the 5 cm DBH class after logging. Post-harvest increases in mortality were not apparent for any focal species and were hence not included in the management simulation model. Lastly, post-harvest diameter growth was not clearly related to maximum unharvested forest growth, precluding the use of maximum growth as post-harvest growth [7].

**Fig 1:**
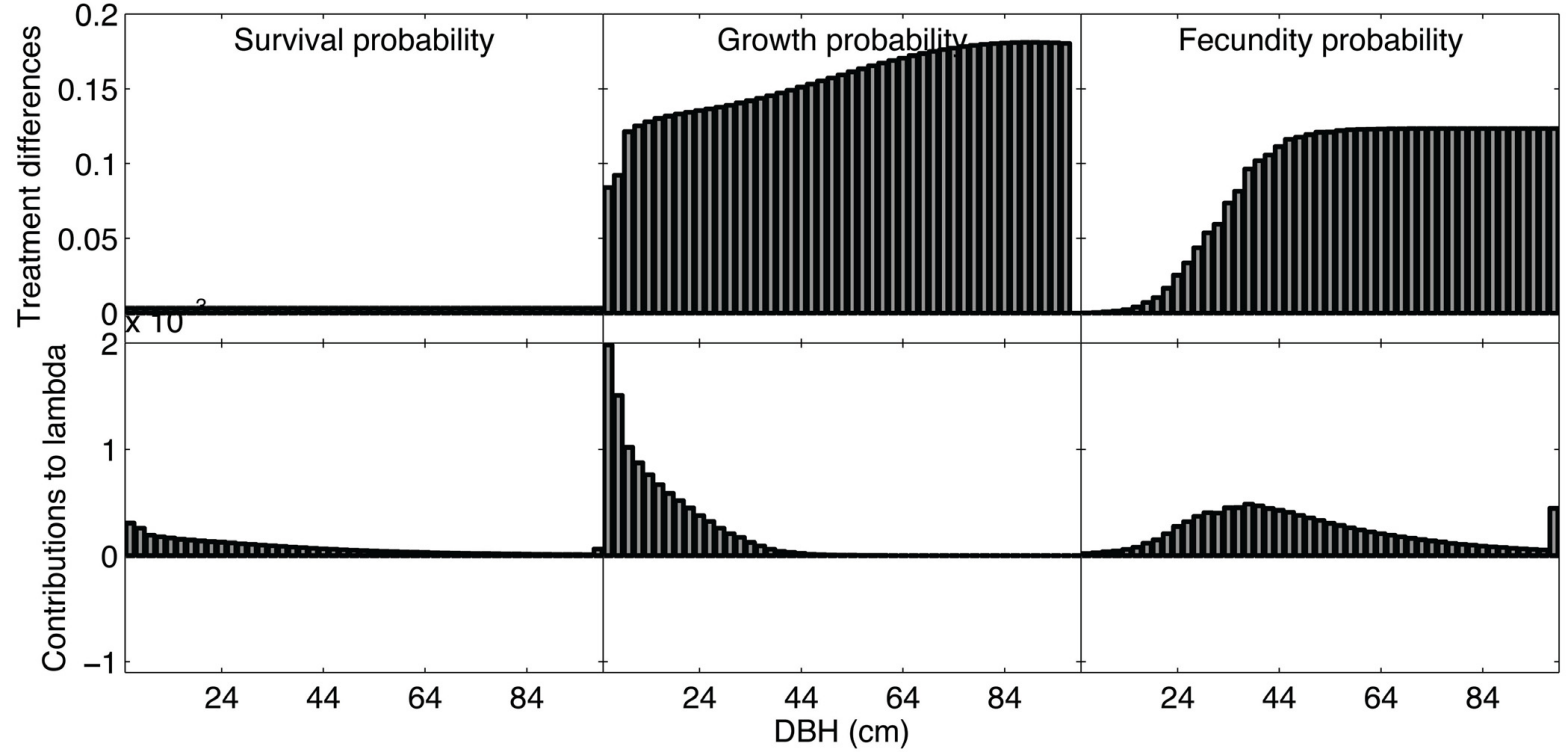
LTRE for *M*. *paraensis* demonstrating demographic differences between harvested and unharvested plots and their contributions to population growth.

Overall, the comparison of population projection matrices using only 2007–2008 data for either unharvested or harvested plots for *M*. *paraensis* and *C*. *spruceanum* (species with sufficient data to parameterize matrices for harvested and unharvested treatments independently) showed that post-harvest λ increases are large for *M*. *paraensis* (1.0272 vs 1.0032; harvested vs unharvested, respectively) but not for *C*. *spruceanum* (0.9944 vs 0.9946; harvested vs unharvested, respectively). The LTRE analysis using these independent harvested/ unharvested matrices shows that the increases in *M*. *paraensis* λ are mostly due to increases in growth of the smaller size classes and increases in fertility by the middle size classes, despite larger responses observed in the larger size classes (Fig 1).

### Harvest damage and mortality

Observed residual stand damage was small and mostly affected palms and non-timber species (Table 3). Observed harvest-induced damage and mortality was minimal for trees >35 cm DBH. For each m^2^ of basal area harvested, an additional 0.11 m^2^ of basal area from timber trees >35 cm DBH was removed due to damage and mortality from felling. Most notably, there was no observed damage or mortality of trees DBH >35 cm due to transport related activities.

**Table 3:** Residual stand damage from monitored timber extraction in terms of basal area (m^2^) of trees >5 cm DBH killed by basal area (m^2^) of timber extracted.

	Tree fall	Transport	Total
**Palm**	0.17	0.03	0.2
**Timber**	0.13	0	0.13
**Other woody spp.**	0.28	0	0.28
**Total**	0.58	0.04	0.62

### Management simulation outputs

Model outputs show that under the management requirements of Brazilian law, on average 50.9 m^3^ of merchantable timber is available per ha in the recently undisturbed forests we sampled. Of this volume, 30 m^3^ could be available for harvest under a 30 yr cutting cycle and 10 m^3^ under a 10 yr cycle following Brazilian law. Model projections indicate fast stock recovery allows continued extraction of maximum allowed volume in subsequent harvests. However, the harvest yields per species change considerably across harvests. Using the 30 year Brazilian legal management regime, *Mora paraensis*, which accounts for 87% of the first harvest, yields approximately 50% of the volume (15 m^3^) for subsequent harvests. Similarly, *Callycophyllum spruceanum* yields 9% of the first harvest volume but by the fifth harvest yields 4% of total harvest volume. On the other hand, yields of historically harvested *C*. *guianensis* and *V*. *surinamensis* increase from 2% (0.5 m^3^) during the first harvest to 23% (7 m^3^) during the fifth harvest for the two species combined.

**Fig 2:**
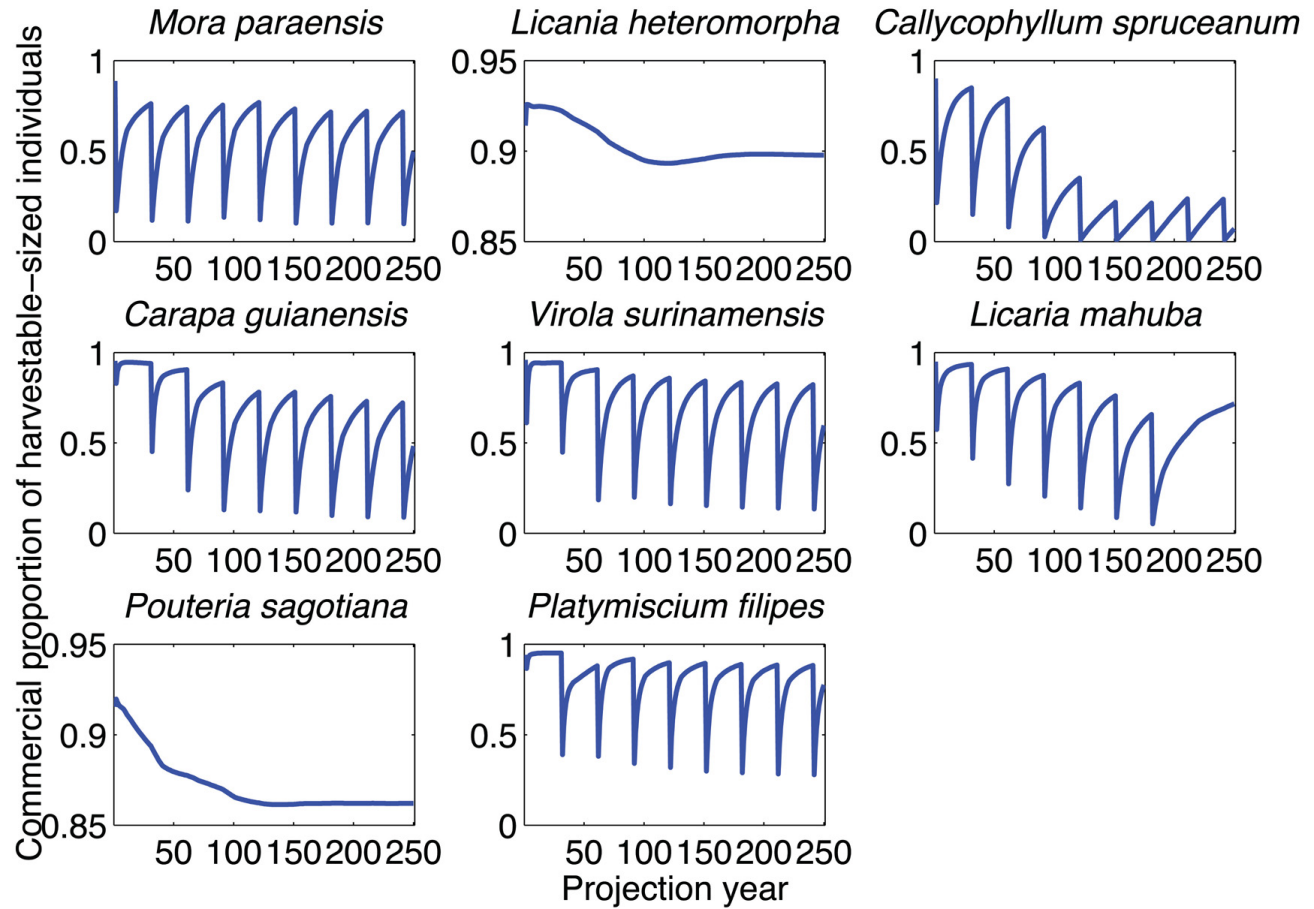
Shifts in merchantable proportion under the 30 yr Brazilian legal management regime but without volume-based harvest limits. *Licania heteromorpha* and *P*. *sagotiana* were not harvested during simulations.

The exclusion of volume-based harvest limits generally increased the rate of decline of merchantable proportion in modeled projections. For instance, without volume-based harvest limits the 30 yr Brazilian legal management regime led to a decrease in the merchantable proportion for all harvested species, but with degree of recovery related to λs (Fig 2). The rapid population turnover of species with high λs results in the stabilization of merchantable proportion at high levels before each simulated harvest (e.g., *V*. *surinamensis* and *P*. *filipes*). In contrast, species with low λs have a low ability to recover between harvests and therefore have merchantable proportions that drop after each harvest (e.g., *C*. *spruceanum* and *L*. *mahuba*). The remaining species with λs slightly above 1 stabilized at pre-harvest merchantable proportions of approximately 0.6–0.7.

The most intensive management regime resulted in the largest first harvests (87 m^3^ with a MCD of 30 cm DBH, 0.9 harvest intensity, and no harvest volume restrictions) but also resulted in very low future harvest volumes (6.2 m^3^ per ha at fifth harvest under a 30-yr cutting cycle). The optimal management regimes for the four STM indicators varied in terms of most management criteria except for the common prescription of short 10-year cutting cycles and, surprisingly, no volume limits per harvests (Table 4). Nearly all optimal management regimes for all STM indicators (except those based on the highest H_3_ AY indicator) included MCDs of 50 cm DBH or greater. Most of the top ranked AY and λ_H_ management regimes required a MCD of 60 cm DBH and volume harvest limits. These STM regimes showed little indication of yield decreases over time and required little additional recruitment to compensate for population growth decreases. All optimal management regimes for the other three STM indicators showed signs of declining yields and higher compensatory recruitment needs. The Brazilian legal management regimes resulted in lower mean AY than any of the optimal STM regimes while still requiring similar numbers of compensatory recruitment, indicating a sub-optimal resource utilization.

**Table 4:** Optimal management regimes defined by alternative sustained timber yield indicators. * Management criteria fixed based on optimal value from analysis of all species combined.

			Optimal criteria					Management outcomes			
Management optimized for	Considering	Cutting cycle	Harvest intensity	MCD	Min. com. density	Seed tree quality	Max volume per harvest	Volume of first harvest	Volume of fifth harvest	Comp. recruitment	Mean AY
Largest mean AY	All species	10	0.5	50	0.03	Low	300	28.94	19.34	10	1.8
Largest H_5_ AY	All species	10	0.9	50	1	Low	300	49.91	21.23	12	1.55
Largest H_3_ AY	All species	10	0.5	40	0.03	Low	300	40.35	16.16	20	1.47
Largest ranked AY and λ_H_	All species	10	0.5	60	1	High	300	15.77	15.02	4	1.44
Legal management regime	All species	30	0.9	50	0.03	Low	30	30	30	22	1
Legal management regime	All species	10	0.9	50	0.03	Low	10	10	10	3	1
Largest ranked AY and λ_H_	*M*. *paraensis*	10*	0.5	60	1	Low	300*	18.02	15.91	5	1.52
Largest ranked AY and λ_H_	*C*. *spruceanum*	10*	0.6	60	1	High	300*	18.92	15.66	5	1.48
Largest ranked AY and λ_H_	*C*. *guianensis*	10*	0.8	60	1	Low	300*	28.84	14.50	5	1.48
Largest ranked AY and λ_H_	*V*. *surinamensis*	10*	0.6	50	1	Low	300*	33.27	17.50	10	1.71
Largest ranked AY and λ_H_	*L*. *mahuba*	10*	0.5	50	0.03	Low	300*	28.94	19.34	10	1.8
Largest ranked AY and λ_H_	*P*. *filipes*	10*	0.9	50	1	Low	300*	49.91	21.23	12	1.55

For each STM indicator we evaluated the differences in optimum management criteria among species. Given the inter-specific demographic differences, there was nearly no overlap among the best species-specific management regimes according to the four STM indicators (S2 File). The inclusion of species-specific regimes led to better results compared to general optimal management regimes (Table 5). The best species-specific management regimes for highest H_5_ AY resulted in a 0.34 m^3^/yr annualized yield gain. The best species-specific management regimes for highest mean AY resulted in a 0.12 m^3^/yr annualized yield gain.

**Table 5:** Differences in annualized yields (m^3^ yr^-1^ ha^-1^) between species-specific and general optimal management regimes under two STM indicators.

	Mean AY	H_5_ AY
*M*. *paraensis*	0.03	0.11
*C*. *spruceanum*	0.02	0.04
*C*. *guianensis*	0.05	0.03
*V*. *surinamensis*	0.01	0.01
*L*. *mahuba*	0	0.14
*P*. *filipes*	0	0
Total	0.12	0.34

### STM sensitivity to management criteria

**Fig 3:**
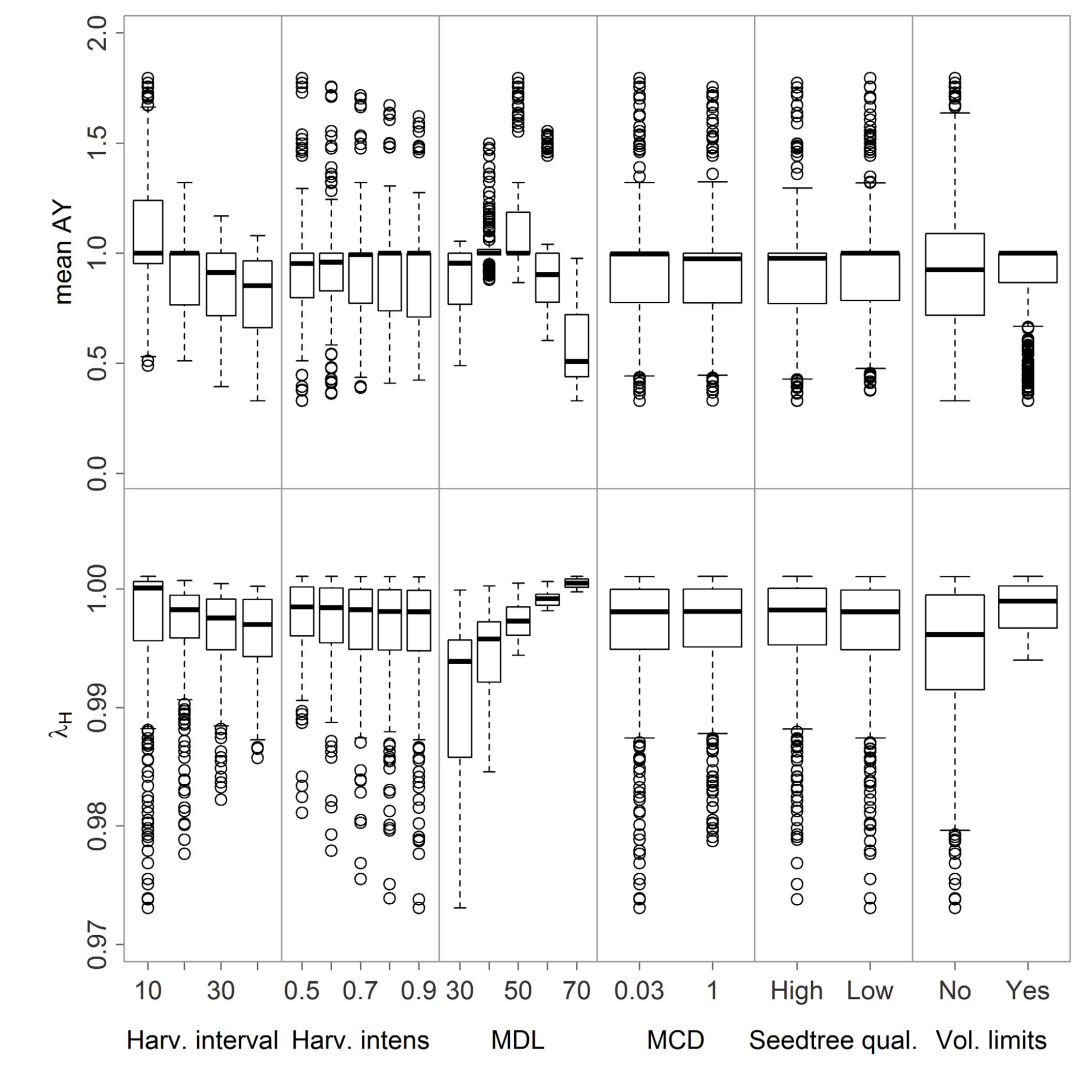
Simulation-based spread of mean AY and λ_H_ in response to varying management criteria.

Stand mean AY decreases with longer cutting cycles and the inclusion of harvest volume limits, but peaks at a MCD of 50 cm (Fig 3). While other management criteria were not significantly correlated to stand mean AY (harvest intensity, minimum density, and seed tree quality), species-specific analyses yielded more nuanced results. Longer cutting cycles lead to large mean AY reductions in *M*. *paraensis*, but also to small increases to *V*. *surinamensis*. Harvest intensity had a small negative impact on mean AY for slow-growing species (*M*. *paraensis*, *C*. *spruceanum*, and *L*. *mahuba)*. Higher MCD has a small positive effect on mean AY of *M*. *paraensis*, but strong effects on all other species. However, this relationship was curvilinear with mean AY peaking around a MCD of 50–60 for most species. Higher MD decreases the mean AY of the least common species (*C*. *spruceanum*, *L*. *mahuba*, and *P*. *filipes*), with its negative effects increasing with species scarcity. Most species-specific λ_H_ increase with higher MCD and the inclusion of harvest volume limits but also suffer mild decreases with increasing harvest intensity. MD has a positive effect on λ_H_ for the three least common species.

## Discussion

### Demography differences between harvested and unharvested estuarine forests

Results from monitoring of harvesting operations show that demographic consequences of selective harvesting were small and likely a result of some damage from non-mechanized operations in relatively vine-free forests. The observed weak harvest effects are in agreement with upland forest studies that show tree fall gaps have small and short-lived demographic effects [17,19]. Crown illumination evaluations and field observations suggest canopy gaps close quickly after logging in the study area, which agrees with assessments of felling damage in upland forest [34,37]. In fact, the unexpected lack of growth response by *C*. *spruceanum* (a light-demanding species) may be due to relatively small canopy openings that benefited its shade tolerant competitors [38]. Logging damage seems an unlikely explanation of the small growth response in *C*. *spruceanum* because damage assessment from harvest monitoring and wider observations from recently logged areas showed minimal damage affecting species indiscriminately.

While other studies have found increased post-harvest mortality persisting years after initial harvests ([39,40]; *but see* [33]), mortality rates in harvested plots did not differ from those observed in unharvested plots in this study. The lack of mortality effect could be partially explained by the observed low residual stand damage, and relatively small gaps that close quickly. Another possibility may be that, unlike unlogged mature stands elsewhere that may contain many structurally weak trees sheltered by a closed canopy [41], the long history of logging may have already culled large structurally-compromised trees in previous harvests.

Widespread advanced regeneration of *Mora paraensis* produced a strong increase in recruitment into the smallest measured size class (>5 cm DBH) after logging for that species alone. Because harvested plots varied from 1 to 6 years since last extraction, it is possible that the post-harvest recruits of other species had not yet reached measurement size. Nevertheless field observations also show there were nearly no *C*. *spruceanum* saplings present in logged and natural gaps, indicating the common extent of logging disturbance is likely insufficient for the recruitment of this and other light-demanding species. Low intensity harvests have been found elsewhere to be insufficient to boost regeneration of light-demanding species [42].These results indicate that the common post-logging challenge to promote regeneration of light-demanding species and to simultaneously reduce logging damage extends to the Amazonian floodplain [3,17,43].

Using *M*. *paraensis* (the most abundant species) as an example, the integration of demographic effects of logging using life table response experiments (LTRE) shows that demographic rates most affected by logging may not be the most important for determining post-harvest tree population dynamics. These conclusions are important because harvest evaluations commonly consider growth, survival, and recruitment effects separately, and may misrepresent changes in population dynamics resulting from observed demographic effects [36]. Without the use of an integrated population approach, however, it would not be possible to detect the larger contribution that smaller juvenile growth increases offer to the persistence of the species in the stand.

### Harvest damage from non-mechanized small-scale logging

Residual stand damage was relatively low compared to other studies [44–47], with no observed damage to large trees during yarding operations. These results bode well for future harvests as these larger trees will constitute the second harvest cohorts. This low residual stand damage is likely a consequence of several favorable factors including the low abundance of vines and lianas that hinder directional felling and cause multiple tree falls, the large abundance of palms which may provide ‘safe’ felling zones [15], the lack of heavy machinery use, and the absence of road construction. A small proportion of residual stand mortality resulted from the need for transport rails and float wood used in the manual transport of timber from the forest, causes of harvest mortality not observed in upland logging operations. These practices avoid use of timber species and instead use species of little to no economic value (e.g., *Inga* species and palms; [25]).

### Prospects for sustainable timber yield

Most evaluations of stock recovery rates in tropical forests are below 50% based on estimated yields following a first harvest, casting doubt over the feasibility of long-term STM [5,7,48,49]. However, results from this study suggest sustaining future harvests is possible in the Amazon Estuary, with the prospects for STM in the studied stands clearly higher than for many other areas evaluated elsewhere in the Amazon [5,7].

In this study, recovery rates between harvests are high for most simulated management regimes due to the comparatively high projected mean AYs [50] that are close to the maximum estimated tropical forest productivity limits [51]. These large mean AYs are primarily due to projected increases in yields from the two fast-growing species *V*. *surinamensis* and *C*. *guianensis* that effectively counterbalance the projected yield declines from other species. Given the historically high volumes of *C*. *guianensis* and *V*. *surinamensis* extracted in the past, the projected recovery of these two species may be explained by the differences between common extractive practices and simulated forested management. As described elsewhere, smallholders often practice re-entry logging where tree populations are harvested down to small tree diameters [10,52]. In fact, model projections show that the harvesting of all merchantable individuals down to small diameters will lead to rapid population and yield declines for both *C*. *guianensis* and *V*. *surinamensis*.

While our results point to increases in the proportion of unmerchantable trees due to consecutive harvests, model results also suggest that fast population growth and related fast diameter growth reduce the possibility of forests of unmerchantable trees. Nevertheless slow-growing species exhibit slow recovery after logging and face continuously decreasing proportion of merchantable trees. For instance, while most study species under simulated management reached a new lower proportion of merchantable trees under intensive harvests, the two species that showed signs of population decline, *C*. *spruceanum* and *L*. *mahuba*, not only exhibited limitations in population recovery but had continuously declining proportions of merchantable trees after each harvest cycle. Fortunately, the number of recruits needed to maintain population stability was low. This need for additional recruits is particularly relevant to the Amazon estuary because, at the smallholder scale of management, additional costs related to managing timber species regeneration are low due to low opportunity costs [53]. Local initiatives to improve seedling supply are underway, but a better understanding of enrichment planning may be needed to ensure success [54]. *Callycophyllum spruceanum's* lack of post-harvest recruitment, despite its ample regeneration in large forest clearings and secondary forests [55] and commonly observed cohort-like size distributions [16] indicates this species is a long-lived canopy pioneer species heavily dependent on large scale disturbances [56]. In fact, widely practiced shifting agriculture decades ago may have benefited this species [13,57]. The prospects for STM of this species would likely be best evaluated by considering how population dynamics of the species interacts with landscape dynamics of the region.

Admittedly, future research could refine our management simulation model with better estimates of post-harvest effects given our relatively small plot area in recently harvested areas. However, it is extremely challenging to collect information from informal, unplanned (and often illegal) micro-scale operations. The collection of the data presented was only possible after multiple years of engagement with the local community, which is an effort that hardly could be scaled up with ease. Yet, currently there are no comparable studies of the long-term impact of timber management available to help refine the broad federal management regulations enforced in the region today.

### Determining best management regimes for sustainable timber management

For Amazonian tidal floodplain forests, general country-level management guidelines provide neither the highest yields nor the highest sustained population growth for species under management. In fact, the variability and complexity of population responses to logging observed here and elsewhere suggest limited prospects for simple and effective one-size-fits-all management prescriptions [5,58]. Due to contrasting species demography, the importance of management practices on yield and population growth varied among species. In some cases even widely accepted management criteria had contrasting effects across species (e.g., harvest volume limits). This complexity of species-specific responses was hidden in stand-level analyses. For instance, analysis with all species combined showed minimum density limits barely affected yield and population growth, as noted elsewhere [49]; However, the same analysis at a species level show this variable is important in terms of yield and population growth for the few rare species included in the analysis.

While we cannot easily implement different harvest intervals for individual species or species groups due to logistical and economic constraints, we can more readily alter species-specific harvest intensity and MCDs. The importance of MCDs to most species future yield and population growth, and the ease of implementing species-specific MCDs suggest that biologically meaningful and species-specific management criteria may provide a feasible STM strategy [49]. A post-hoc analysis for optimal species-specific management regimes under the ranked AY and λ_H_ indicator holding the cutting cycle length and harvest volume limits constant at the optimal stand values results in easily implementable species-specific management regimes (Table 4). Across all STM indicators, nearly all optimal STM regimes include MCDs ≥ 50 cm DBH, which bodes well for current Brazilian legislation with similar requirements. A central factor in determining yields of the Brazilian legal management regimes is the inclusion of harvest volume limits. This study shows that such an overarching rule may in some cases lead to resource underutilization. In fact, the mean AY ∩ λ_H_ optimal management regime produces a much higher mean AY with nearly no need for extra compensatory recruitment. However, on a cautionary note, our results show how optimal STM regimes partly depend on how STM is quantified. STM indicators with potentially short management horizons, such as the H3 and H5 AY indicators, led to aggressive optimal management regimes that still resulted in large drops in harvest volume between first and later harvests. Overall, ranked AY and λ_H_ seems to be the best performing STM indicator as it prevents decreases in harvest volumes, guarantees high mean AY while requiring the least number of compensatory recruits per harvest of the four indicators used.

Our research shows that, using population modeling tools, it is possible to develop management guidelines that are specific to a region’s ecological settings and that can be further fine-tuned to consider differences in species demography and differential responses to repeated harvests. As such, our optimal STM regimes offer a first approximation of guidelines that may better balance yield and population growth concerns related to tropical STM. Much of the focus on the very limited advance in Tropical STM has been on the widespread disregard of most timber operations to management standards and guidelines. In principle, ecologically fine-tuned management guidelines could make management more attractive and thus help bridge the gap between practice and guidelines that is currently prevalent in the tropical timber industry.

## Supporting Information

S1 File Description of grouped density dependence modeling approach and results. (PDF)

S2 File Comparison of species-specific optimal management regimes according to four STM indicators. (PDF)
