# Supplementary material for: Modeling the Complex Impacts of Timber Harvests to Find Optimal Management Regimes for Amazon Tidal Floodplain Forests
Source: PLoS One. 2015 Aug 31;10(8):e0136740. doi: 10.1371/journal.pone.0136740 (PMC4556458; doi:10.1371/journal.pone.0136740)
Supplement: S1 File — (PDF) [file pone.0136740.s001.pdf]

APPENDIX 1. DESCRIPTION OF GROUPED DENSITY DEPENDENCE MODELING APPROACH AND RESULTS

Unlike the standard approach of incorporating population regulation by modeling density dependence and applying it for individual species, we experimentally replicated our analyses where density dependence regulation occurs based on overall stand density and not individual species densities. This was simply done by fitting our Weibull density dependence function based on stand density instead of species by species.

$$Recruitment_{Na} = a \times exp(-Na/b)^{6}$$

This species-grouped K and Na model parameterization allowed for the competitive replacement of species being harvested or dying naturally by individual of species with ample recruitment, allowing for a simple mechanism for species composition shift after logging.

The model based on grouped-K density-dependence yielded simulation outputs in which the speed of shifts in species relative abundance seemed related to species λs and intensity of management regimes (Figure below). Using a group K allowed for the shift in species composition that is well documented in logging operations elsewhere (Dickinson, Whigham & Hermann 2000; Fredericksen & Mostacedo 2000; Dekker & de Graaf 2003) and merits future research as a potentially useful minimalist model for stand dynamics.

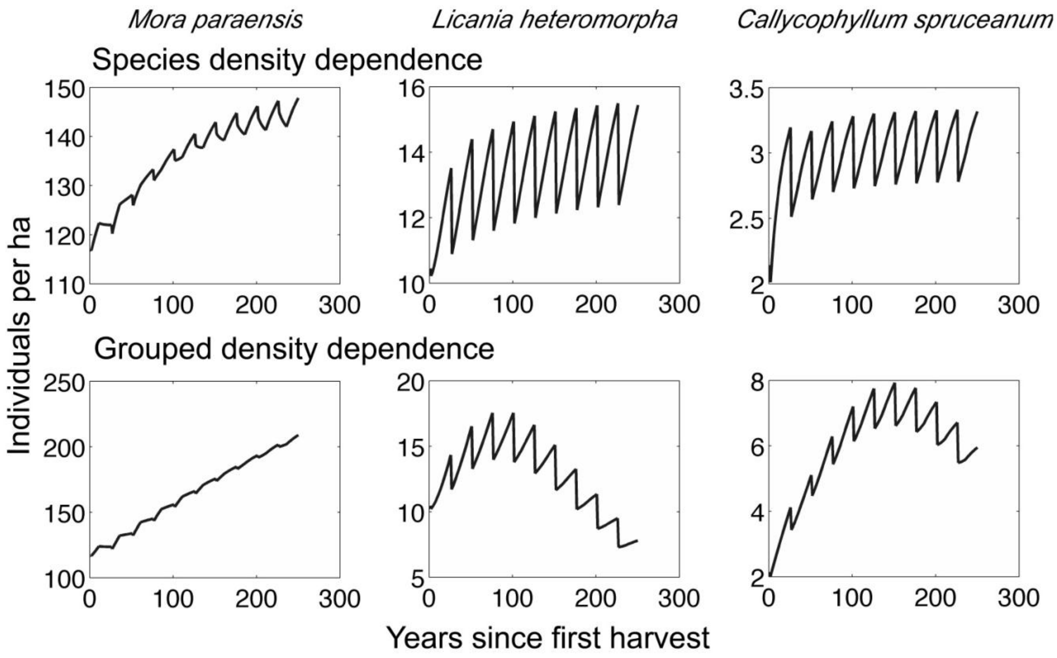

Management simulation projections illustrating differences between species and grouped density-dependent models

Preliminary model results based on applying density dependence using stand level carrying capacity show that forest composition can change considerably over the course of multiple harvest rotations. While few studies explore the consequences of logging on tropical forest composition, those that address it generally conclude these changes can affect long-term ecological and economic viability of yields (Alder & Silva 2000; Phillips *et al.* 2004).

Literature cited:

Alder, D. & Silva, J.N.M. (2000) An empirical cohort model for management of Terra Firme forests in the Brazilian Amazon. *Forest Ecology and Management*, **130**, 141–157.

Dekker, M. & de Graaf, N.R. (2003) Pioneer and Climax Tree Regeneration Following Selective Logging With Silviculture in Suriname. *Forest Ecology and Management*, **172**, 183–190.

Dickinson, M., Whigham, D. & Hermann, S. (2000) Tree regeneration in felling and natural treefall disturbances in a semideciduous tropical forest in Mexico. *Forest Ecology and Management*, **134**, 137–151.

Fredericksen, T. & Mostacedo, B. (2000) Regeneration of timber species following selection logging in a Bolivian tropical dry forest. *Forest Ecology and Management*, **131**, 47–55.

Phillips, P.D., De Azevedo, C.P., Degen, B., Thompson, I.S., Silva, J.N.M. & Van Gardingen, P.R. (2004) An Individual-based spatially explicit simulation model for strategic forest management planning in the Eastern Amazon. *Ecological Modelling*, **173**, 335–354.
