# Supplementary material for: Modeling the Complex Impacts of Timber Harvests to Find Optimal Management Regimes for Amazon Tidal Floodplain Forests
Source: PLoS One. 2015 Aug 31;10(8):e0136740. doi: 10.1371/journal.pone.0136740 (PMC4556458; doi:10.1371/journal.pone.0136740)
Supplement: S2 File — (PDF) [file pone.0136740.s002.pdf]

APPENDIX 2. COMPARISON OF SPECIES-SPECIFIC OPTIMAL MANAGEMENT REGIMES ACCORDING TO FOUR STM INDICATORS.

Ones denote at least one match in the top three optimal management prescriptions between species, zeros a mismatch.

	M. paraensis	*C. spruceanum*	*C. guianensis*	*V. surinamensis*	*L. mahuba*	*P. filipes*
Largest mean AY						
M. paraensis	1					
C. spruceanum	0	1				
C. guianensis	0	0	1			
V. surinamensis	0	0	1	1		
L. mahuba	0	0	0	0	1	
P. filipes	0	0	0	0	1	1
Largest H_5 AY						
M. paraensis	1					
C. spruceanum	1	1				
C. guianensis	1	1	1			
V. surinamensis	1	1	1	1		
L. mahuba	0	0	0	0	1	
P. filipes	0	0	0	0	0	1
Largest H_3 AY						
M. paraensis	1					
C. spruceanum	0	1				
C. guianensis	0	1	1			
V. surinamensis	0	1	1	1		
L. mahuba	0	0	0	0	1	
P. filipes	0	0	0	0	0	1
High mean AY ∩ λH						
M. paraensis	1					
C. spruceanum	0	1				
C. guianensis	1	1	1			
V. surinamensis	0	0	0	1		
L. mahuba	0	0	0	0	1	
P. filipes	0	0	0	1	0	1
